# Passive Extraction of Signal Feature Using a Rectifier with a Mechanically Switched Inductor for Low Power Acoustic Event Detection

**DOI:** 10.3390/s20185445

**Published:** 2020-09-22

**Authors:** Marko Gazivoda, Dinko Oletić, Carlo Trigona, Vedran Bilas

**Affiliations:** 1 Faculty of Electrical Engineering and Computing, University of Zagreb, 10000 Zagreb, Croatia; dinko.oletic@fer.hr; 2 Department of Electrical, Electronic and Computer Engineering, University of Catania, 95100 Catania, Italy; carlo.trigona@dieei.unict.it

**Keywords:** wake-up interface, acoustic vibrations, pattern recognition, low SNR, passive electromechanical transducer

## Abstract

Analog hardware used for signal envelope extraction in low-power interfaces for acoustic event detection, owing to its low complexity and power consumption, suffers from low sensitivity and performs poorly under low signal to noise ratios (SNR) found in undersea environments. To overcome those problems, in this paper, we propose a novel passive electromechanical solution for the signal feature extraction in low frequency acoustic range (200–1000 Hz), in the form of a piezoelectric vibration transducer, and a rectifier with a mechanically switched inductor. A simulation study of the novel solution is presented, and a proof-of-concept device is developed and experimentally characterized. We demonstrate its applicability and show the advantages of the passive electromechanical device in comparison to the active electrical solution in terms of operation with lower input signals (<20 mV compared to 40 mV), and higher robustness in low SNR conditions (output voltage loss for −10 dB ≤ SNR < 40 dB of 1 mV, compared to 10 mV). In addition to the signal processing performance improvements, compared to our previous work, the utilization of the presented novel passive feature extractor would also decrease power consumption of a detector’s channel by over 76%, enabling life-time extension and/or increased quality of detection with larger number of channels. To the best of our knowledge, this is the first solution presented in the literature that demonstrates the possibility of using a passive electromechanical feature extractor in a low-power analog wake-up event detector interface.

## 1. Introduction

The recognition of infrequent events is of interest in many fields (environmental monitoring [1,2,3,4,5], safety and security [6,7,8,9,10,11,12,13], communication [14,15,16], agriculture, health monitoring [17]). It requires continuous operation of an electronic system comprising sensing, detection and recognition functions, which are power-hungry tasks [7,18]. The power consumption can be reduced by introduction of always-on low-power interfaces that wake up the main processing stage upon detection of an event of interest [19,20].

**Figure 1:**
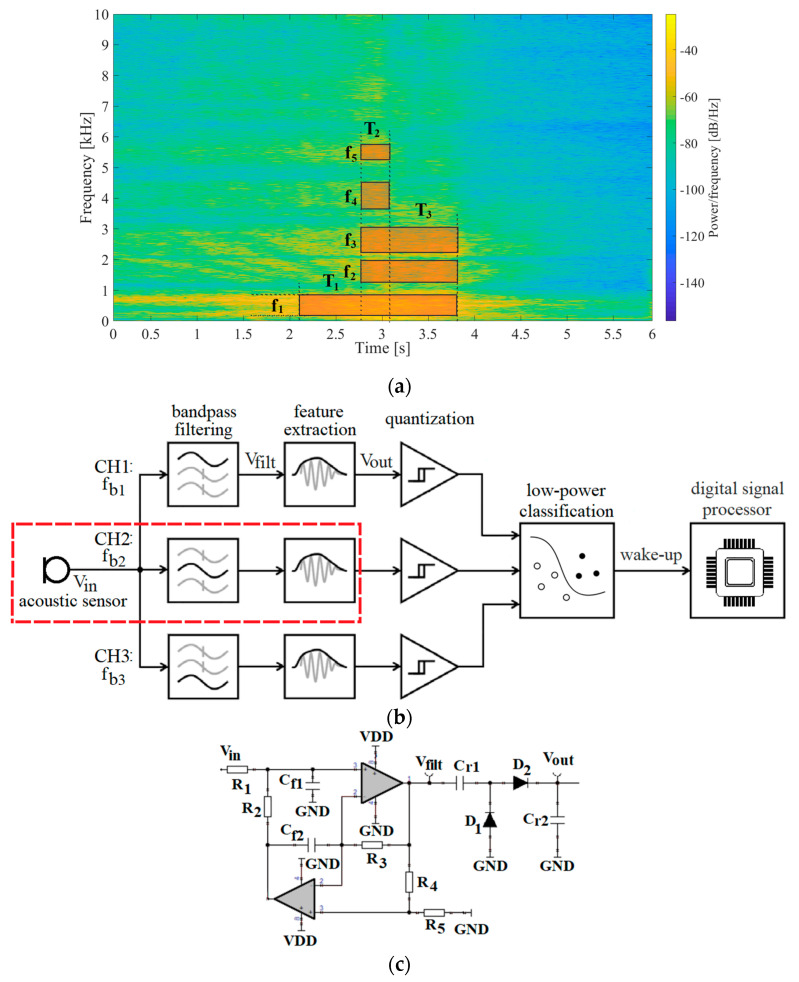

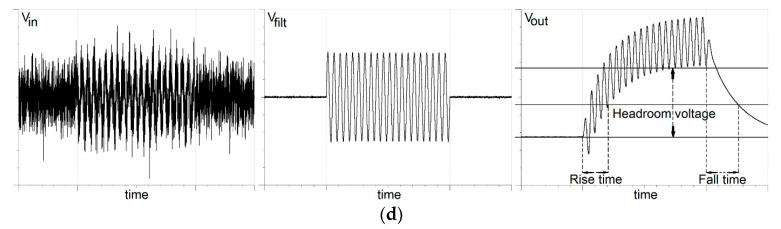
(**a**) An example of a spectrogram of a signal of interest. *T*_1_, *T*_2_, *T*_3_, represent durations, *f_b_*_1_, *f_b_*_2_, *f_b_*_3_ frequency bands and *E*_1_, *E*_2_, *E*_3_ energy (feature) of states within the time-frequency pattern. (**b**) Block diagram of an analog multichannel pattern recognition-based event detection system. The marked part of the system is the active electrical feature extractor (in this case the feature is signal envelope in a set frequency band). (**c**) A schematic of the active electrical feature extractor (AE FE). (**d**) Characteristic signals of the detector—at transducer (*V_in_*), after filter (*V_filt_*) and at output (*V_out_*). The values of interest in the feature extractor’s output voltage are marked—headroom voltage, rise and fall time.

Many acoustic events can be recognized based on their time-frequency pattern [5], which can be approximated by an ordered sequence of discrete time-frequency states (Figure 1a). The extraction of these patterns in wake-up systems is usually performed by processing in the time domain utilizing a low-power multichannel analog detector [21]. The event detector consists of multiple channels to allow for simultaneous analog frequency decomposition, with each channel requiring a feature extractor to extract features from a designated frequency band from the signal of interest. To make the detector simple and efficient it is crucial to choose an appropriate feature and its extraction method. Features that can be used in these detectors can either be instantaneous ones (envelope) or related to the signal integral (root mean square (RMS), power, energy) [22,23,24]. Most of low-power event detection systems use instantaneous signal features because of their simple extraction (shown in Figure 1b). In our previous work [25], we developed such a system, with a power consumption of 11.52 µW per channel. In this work, we will focus on the feature extraction part of the acoustic event detection system.

Despite the envelope having the advantage of being one of the simplest features to extract in terms of hardware complexity and power consumption, a serious drawback of the envelope is its poorer performance in low signal to noise ratios (SNR) conditions [26].

Following an idea of using electromechanical components to improve low-power event detector functionality [20], our goal in this work was to evaluate a passive electromechanical feature extractor that utilizes a piezoelectric transducer and a rectifier with a mechanically switched inductor, inspired by the Random Mechanical Switching Harvester on Inductor (RMSHI) [27,28,29], as an alternative for active electrical feature extractor (Figure 1c), consisting of an active bandpass filter and a passive rectifier used for detecting slowly evolving acoustic events in low SNR conditions in frequency range from 200 Hz to 1 kHz.

In [30], we analyzed the applicability of a rectifier with a mechanically switched inductor (part of RMSHI) used with an active bandpass filter forming an active feature extractor. The goal of this work was to develop and characterize a novel, fully passive electromechanical feature extractor (PEM FE) implementing a transducer, a mechanical filter and a rectifier with a mechanically switched inductor, to be utilized in an acoustic event detection.

In this paper we make several contributions. We present a simulation model of the passive electromechanical feature extractor as part of an acoustic event detector and present simulation results. We develop a proof-of-concept extractor and, with it, provide experimental verification of the simulation results. We demonstrate applicability and advantages of the extractor in extraction of features under low SNR. To the best of our knowledge, this is the first solution presented in the literature that shows a low-power acoustic event detector utilizing a passive electromechanical feature extractor. This work represents a step towards a fully passive, or self-sustaining, infrequent acoustic event detector.

The rest of the paper is organized as follows: Section 2 shows an event detector architecture, describing the passive electromechanical feature extractor. Section 3 shows the simulation model and simulation results of the passive electromechanical feature extractor. Section 4 shows experimental results and characterization of the developed extractor. Section 5 presents comparison of an active electrical and passive electromechanical feature extractor. Section 6 concludes the paper and shows future research.

## 2. Event Detector Architecture

### 2.1. Time-Frequency Signal Pattern and Event Detection System

A spectrogram of a passing ship with marked time-frequency states and a pattern recognition-based event detector are shown in Figure 1a,b, respectively [25]. The detector entails three analog-domain filtering channels decomposing the transducer’s signal (*V_in_*) into arbitrary frequency bands. Intervals of signal presence within individual filtering channels (*V_filt_*) are localized in time by extracting features of filtered signals, and then performing single-bit quantization using comparators. Temporal relations between comparator responses are finally analyzed by a 3-channel digital sequence recognition state-machine, which wakes-up the digital signal processor DSP [21]. A schematic of detector’s active electrical feature extractor (AE FE) is shown in Figure 1c.

Detailed characterization of this detector utilizing an active general impedance converter (GIC)-based dual operational amplifier bandpass filter was performed in [25] and a study on rectifier topologies for envelope extraction was presented in [31]. Such a combination of an active bandpass filter and passive diode rectifier developed in previous work showed several constraints on this detector’s feature extractor performance and output signal (Figure 1d). The rise and fall times determine the detector’s ability to distinguish two consecutive time-frequency states. Based on our previous study, rise and fall times around 1 s are acceptable for slow-evolving acoustic events of interest. The output headroom voltage of the envelope extractor shows variations in the feature of interest. The headroom voltage required for event detection is dependent on the selected comparator. In this work, we consider a level of 1 mV to be acceptable.

In this work, we intend to replace the active electrical feature extractor (AE FE), consisting of an active bandpass filter and a passive rectifier (Figure 1c), with a passive electromechanical feature extractor (PEM FE) that features a rectifier with a mechanically switched inductor (Figure 3), to reduce power consumption and improve performance in high background noise environments.

### 2.2. Passive Electromechanical Feature Extractor

**Figure 2:**
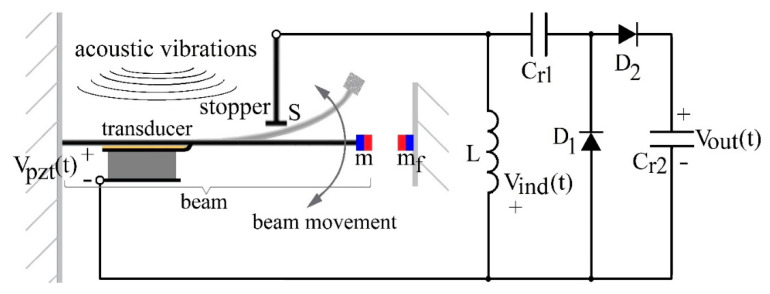
Block diagram of the passive electromechanical feature extractor (PEM FE). *S*—switch, *m*—magnet at the beam end, *m_f_*—fixed magnet, *L*—inductor, *C_r_*_1_, *C_r_*_2_—rectifier capacitors, *D*_1_, *D*_2_—rectifier diodes. *V_pzt_*(*t*)—voltage generated at piezoelectric transducer, *V_ind_*(*t*)—voltage induced at the inductor *L*, *V_out_*(*t*)—extractor output voltage.

The mechanical structure of the proposed passive electromechanical feature extractor (PEM FE) (Figure 2) is based on Random Mechanical Switching Harvester on Inductor (RMSHI). The RMSHI was developed for vibration energy harvesting utilizing a piezoelectric transducer. Its key advantage is high efficiency of converting vibration into electrical energy, which is 0.622%, compared to the 0.022% for resonant harvesters with single-frequency excitation, and 0.126% compared to 0.001% for broad frequency excitation [32]. The greater energy conversion efficiency of the RMSHI stems from: non-resonant frequency response and utilization of a mechanically switched inductor.

The RMSHI consists of a metal cantilever beam having a permanent magnet at its free end, a metal stopper that forms a mechanical switch with the cantilever, and a fixed permanent magnet that modifies elasticity of vibration driven movement of the cantilever. A piezoelectric transducer is mounted on a metal cantilever beam that transforms input vibrations into an electrical signal. An inductor is switched by the cantilever movement and connected to a rectifier that conditions the electric signal (a mechanically switched inductor).

Due to the RMSHI featuring a stopper and a pair of magnets exerting repulsive force on each other (as shown in Figure 2), the movement of the RMSHI cantilever beam can be considered a movement of two beams attached to one another, with an initial magnetic force bias placing the beam in contact with the stopper when no external force is applied. While the beam is in contact with the stopper, only its part in front of the stopper moves. Once the overall external force on beam overcomes the magnetic force bias, the beam detaches from the stopper and the entire beam moves. This two-part movement causes the beam to have a non-resonant, broader frequency characteristic. Further explanation on this can be found in [27,32].

The utilization of the mechanically switched inductor provides further increase in harvester efficiency in conditions of weak vibrations, generating signals under the diode threshold.

**Figure 3:**
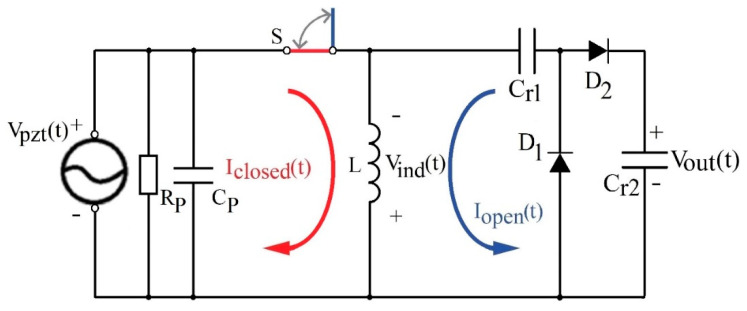
Schematic of the proposed passive electromechanical feature extractor (PEM FE). Red—current *I_closed_*(*t*)—passing through the PEM FE while the switch *S* is closed. Blue—current *I_open_*(*t*) passing through the PEM FE when the switch *S* opens. *L*—inductor and *C_r_*_1_, *C_r_*_2_—rectifier capacitors, *D*_1_, *D*_2_—rectifier diodes. *R_P_* and *C_P_*—parasitic resistance and capacitance of piezoelectric transducer, respectively. *V_pzt_*(*t*)—voltage generated at piezoelectric transducer, *V_ind_*(*t*)—voltage induced at the inductor, *V_out_*(*t*)—extractor output voltage.

When the beam and the stopper are in contact, the switch *S* is closed, and the energy of the signal generated by the piezoelectric transducer is stored in the inductor’s magnetic field (Figure 3, red details). When the switch opens, the energy stored in the inductor’s (*L*) magnetic field, *E_L_*, is transferred over the rectifier to the capacitor’s (*C_r_*_2_) electric field energy, *E_C_*, charging the capacitor to *V_out_* (Figure 3, blue details). Neglecting energy losses, voltage *V_out_* depends on the current through the inductor at that instant of time, *I_open_*, and the values of inductance *L* and capacitance *C_r_*_1−2_ (1) [33]

(1) $$V_{out}=I_{open}\cdot \sqrt{\frac{L}{C_{r1-2}}}$$

The switching of the inductor induces voltage, *V_ind_*(*t*), higher than the voltage generated by the piezoelectric transducer, *V_pzt_*(*t*), and the diode threshold, making the switched inductor a voltage booster. A detailed description of the process is presented in [27,28,32].

The exact physical implementation of the extractor used in this study, with transducer and switch parameters will be presented in the Measurement Setup subsection in Section 4.

## 3. Passive Electromechanical Feature Extractor Simulation Model and Simulation Results

### 3.1. Simulation Model

In order to evaluate the use of the passive electromechanical feature extractor (PEM FE) in a set frequency band, an analytical model has been implemented and simulated in MathWorks’ MATLAB^®^ Simulink (Natick, MA, USA).

The mechanics of the PEM FE can be modeled by a second order non-linear differential equation:

(2) $$mx^{\cdot \cdot }+dx^{\cdot }+{\frac{\partial U_{T}}{\partial x}=F\left(t\right)|}_{U_{T}=\delta x^{4}}$$

where *m* is the mass of the beam with the magnet and *d* is its damping coefficient. Term *x* is the displacement of the tip of the beam and the dotted terms represent the first and second derivate of the displacement (velocity and acceleration of the cantilever, respectively). *U_T_* is the potential energy function, which is non-linear due to the stopper and permanent magnet positions, which act as adjustable factors, allowing fine adjustments within ranges determined by the physical dimensions of the device (beam length, *l* and mass, *m*). The magnet’s contribution is expressed with the term *δ*.

The voltage generated on the piezoelectric transducer (*V_pzt_*(*t*)) on the cantilever beam, is:

(3) $$V_{pzt}\left(t\right)=\int (\Pi \dot{x}-V_{pzt}\left(t\right)/\tau )dt$$

where ∏ is the coupling constant and *τ* is the time constant of the piezoelectric material. In detail, all parameters describing the previous equations are listed and their values, originated by the model, experiments and the literature, are indicated in Table 1.

**Figure 4:**
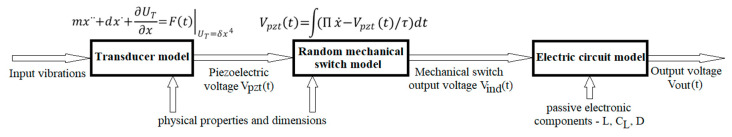
Simplified simulation model of the passive electromechanical feature extractor (PEM FE). It consists of simulation models of the piezoelectric transducer, the mechanical switch and the electric circuit.

The analytical considerations presented by Equations (2) and (3) constitute the basis for the development of the simulation model (simplified version is shown in Figure 4). The topology of the electric circuit used in the model is the same as shown in Figure 3. The inductor *L* used for the simulation model was 1 mH and the diodes were modeled to fit the D1N4148 diode characteristics. The rectifier capacitances, *C_r_*_1−2_, used in the simulations were: 33 nF, 100 nF, 470 nF and 1 µF. The physical parameters of the PEM FE models, beam length (*l*), beam and magnet mass (*m*) and magnet position (*δ*) were varied to achieve different frequency characteristics, while the parameters concerning the piezoelectric transducer (*∏* and *τ*) were fixed to simulate the behavior of the used piezoelectric transducer as closely as possible.

Using the simulation model, the following analyses were done: the relation between the input energy (*E_in_*) and the RMS value of the output voltage (*V_out_*) (transfer characteristic of the passive electromechanical feature extractor), the frequency selectivity of the extractor and the rectifier capacitances’ (*C_r_*_1−2_) influence on the output voltage (*V_out_*) waveform.

### 3.2. Simulation Results

**Figure 5:**
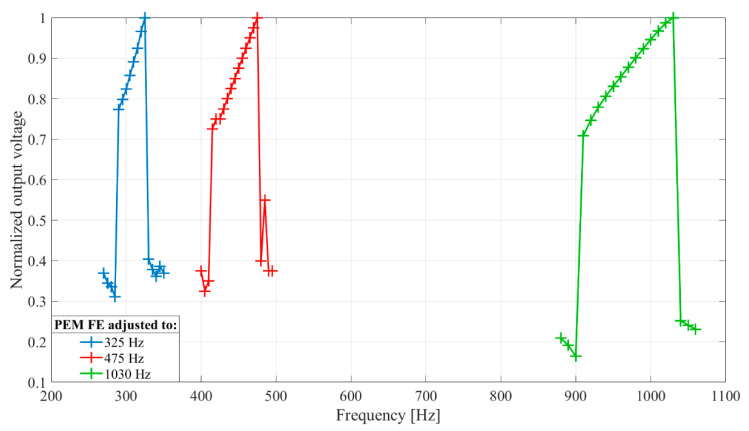
Frequency selectivity of passive electromechanical feature extractor (PEM FE) obtained by change of its physical dimensions (beam length (*l*), beam and magnet mass (*m*), magnet position (*δ*)). The output voltage was normalized with regards to maximal value. Rectifier capacitances *C_r_*_1−2_ = 33 nF. Inductor *L* = 1 mH. Input vibration frequency changed by 5 Hz from 270 Hz to 350 Hz, from 400 Hz to 495 Hz and from 880 Hz to 1060 Hz for each PEM FE setting, respectively. Input vibration energy set to 1.6 nJ.

The simulation analysis of PEM FE frequency selectivity shows that its frequency characteristic can be adjusted to fit a selected band within the lower acoustic frequencies of interest (up to around 1 kHz) by changing its physical parameters explained in Section 3.1 and shown in Figure 4. The results in Figure 5. show three feature extractors for different selected frequency bands. Rectifier capacitances were set to *C_r_*_1−2_ = 33 nF and an inductor of *L* = 1 mH was used. Input vibration frequency changed by 5 Hz from 270 Hz to 350 Hz, from 400 Hz to 495 Hz and from 880 Hz to 1060 Hz for each extractor, respectively. Input vibration energy was set to 1.6 nJ.

**Figure 6:**
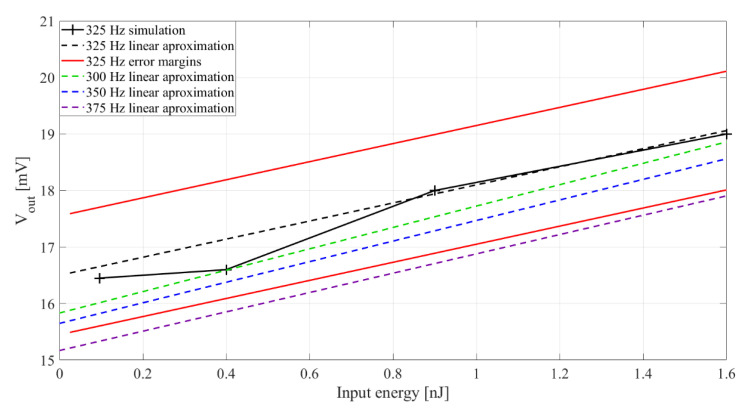
Extractor output voltage (*V_out_*) with input signal energy, *E_in_*. Rectifier capacitance *C_r_*_1−2_= 33 nF. Inductor *L =* 1 mH. Input vibration energy was set to 0.1 nJ, 0.4 nJ, 0.9 nJ and 1.6 nJ, respectively. The black line with pluses represents the simulation results, the black dashed line the linear approximation and the red lines represent the error margins (explained in more detail in the experimental part) for 325 Hz input vibration frequency. The green, blue and purple dashed lines represent linear approximations for 300 Hz, 350 Hz and 375 Hz input vibration frequency, respectively.

The simulation analysis of the relation between input vibration energy and output voltage shows (Figure 6) that a PEM FE can operate with sufficiently high sensitivity of 2 mV/nJ in a frequency band around 325 Hz, and with input vibration levels of interest (from 0.1 nJ to 1.6 nJ), proving it can be used as a feature extractor in an acoustic event detector channel. The measurement resolution of ±1 nJ can be achieved. The analysis also shows the sensitivity with different input vibration frequencies, around the passband central frequency of 325 Hz (dashed lines in Figure 6). Rectifier capacitances were set to *C_r_*_1−2_ = 33 nF and the inductor was set to *L* = 1 mH.

**Figure 7:**
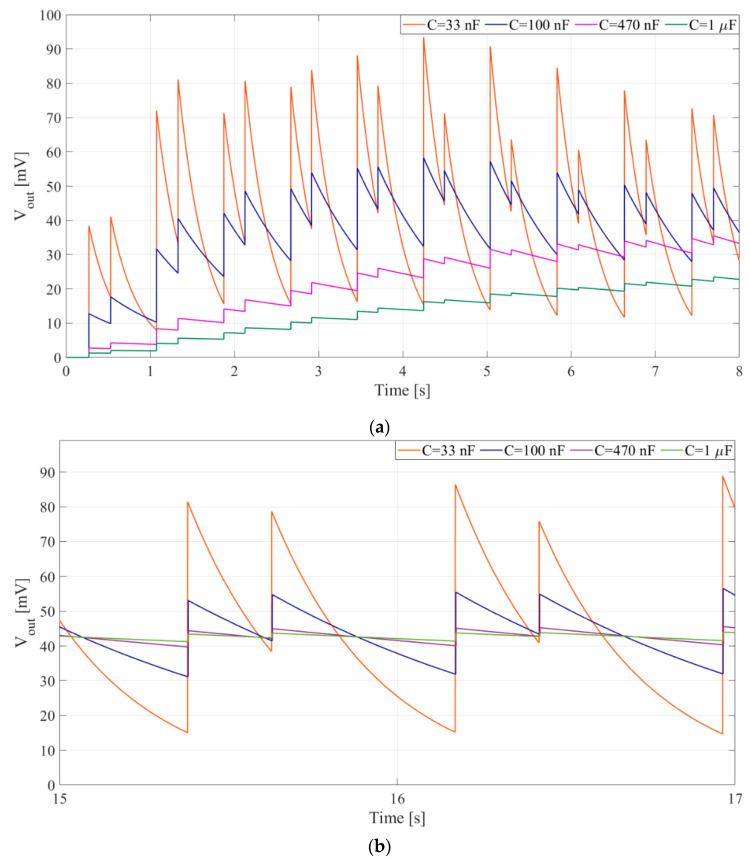
Waveform of the output voltage, *V_out_*, for several values of rectifier capacitances: 33 nF, 100 nF, 470 nF, 1 μF. Input vibrations at 325 Hz generate 50 mV peak-to-peak at the piezoelectric transducer. Inductor *L* = 1 mH. (**a**) At beginning of capacitor charging, (**b**) In stationary conditions.

The simulation analysis concerning rectifier capacitances shows the influence the rectifier capacitance has on PEM FE’s output voltage waveform. For higher values of the rectifier capacitances a decrement in output voltage (*V_out_*) ripple can be observed from around ± 33 mV to around ± 2.5 mV (Figure 7b), increasing the headroom voltage (Figure 1d), but this is counterbalanced with an increment in rise and fall times (Figure 1d) from around 1 s to around 10 s (Figure 7a).

## 4. Experimental Characterization of Passive Electromechanical Feature Extractor

### 4.1. Measurement Setup

The goal of these measurements was to provide experimental verification of the simulation results and characterize the passive electromechanical feature extractor in terms of selectivity and sensitivity and to confirm design parameters.

**Figure 8:**
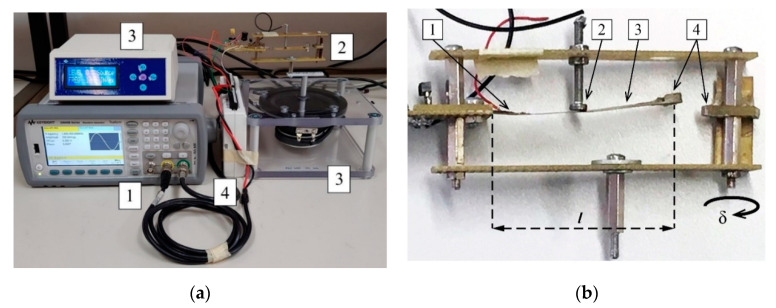
(**a**) A photograph of the measurement setup. (1) Keysight 33500B waveform generator, (2) PEM FE,(3) Smart Material Energy Harvesting Kit 1.2. shaker, (4) NI USB-6211 data acquisition card. (**b**) Physical realization of the PEM FE (without the rectifier). (1) Piezoelectric transducer, (2) stopper, (3) cantilever beam, (4) fixed magnet (right) and adjustable magnet (left). The mass of the beam (*m*) is approximated by the mass of the magnet at its end. *δ* is the magnet position adjustment parameter. Length of the beam is marked by *l*.

A photograph of the measurement setup can be seen in Figure 8a. The measurement setup consisted of a waveform generator (Keysight 33500B) that drives the shaker (Smart Material Energy Harvesting Kit 1.2.) used both to generate the voltage on a piezoelectric transducer and to open and close the switch in the extractor. The output voltage was acquired by a National Instruments data acquisition card (NI USB-6211) connected to a PC.

Figure 8b shows the physical realization of the PEM FE used in this study. The piezoelectric element was bonded to the cantilever in part of the beam where maximal strain was measured. The piezoelectric material used in this study is a 7BB-12-9 piezoelectric diaphragm (Murata), with an external diameter of 12 mm and thickness of 0.22 mm, [34]. The electromechanical switch used in this study is composed of a brass beam with a length (*l*) of about 55 mm, width of about 8.3 mm and thickness of about 0.2 mm, with the distance (*l*_1_) between the anchor of the cantilever and the stopper of about 28 mm. This choice is correlated with an optimization process as shown in [35].

### 4.2. Measurement Procedure

#### 4.2.1. Transfer Characteristics

The goal was to characterize the PEM FE’s output voltage sensitivity to input vibration energy. Rectifier capacitors, *C_r_*_1−2_, are set to 33 nF. The inductor, *L*, of 100 mH was chosen. The extractor was adjusted to have the highest output voltage at frequency of 315 Hz. The frequency of the input signal was 315 Hz, and its energy was set to 0.05 nJ, 0.1 nJ, 0.4 nJ, 0.9 nJ and 1.6 nJ, respectively. The energy level is estimated as external applied force (mass of the extractor multiplied by its acceleration) multiplied by the displacement of the beam [32]. For each input energy level, 10 measurements were done, in duration of 5 s each, and output voltage *V_out_* RMS value was averaged for each energy level. The measurement error *ε* was calculated as:

(4) $$\varepsilon =3A=3\sqrt{\frac{std^{2}\left(RMS1,RMS2,\ldots ,RMS10\right)}{10}}$$

where *A* is the uncertainty and *std* is the standard deviation of the voltage values of the 10 measurements.

#### 4.2.2. Frequency Selectivity

The goal was to determine the relation of output voltage and input vibration frequency, within the selected frequency band. Rectifier capacitors, *C_r_*_1−2_, are set to 33 nF. Inductor, *L*, of 100 mH is chosen. The frequency of the input signal was swept from 150 Hz to 210 Hz with increment of 10 Hz for one developed extractor and from 290 Hz to 330 Hz with increment of 5 Hz for the other. For each input signal frequency, the output voltage was recorded in duration of 20 s. The recorded waveforms were processed in MATLAB^®^ to obtain normalized RMS values of output voltage for each frequency.

#### 4.2.3. Design Parameters

The goal was to select the rectifier capacitors *C_r_*_1−2_ considering output voltage ripple and response times. The frequency of the input signal was set for maximal output voltage (to 315 Hz), and to generate a voltage on the piezoelectric transducer of 100 mV peak-to-peak. Input vibrations were gated, with 0.75 s of signal, followed by 4 s of pause. The output voltage was measured for capacitors of 33 nF, 100 nF, 470 nF and 1 µF. The inductor, *L*, of 100 mH was used for the experiment.

### 4.3. Measurement Results

**Figure 9:**
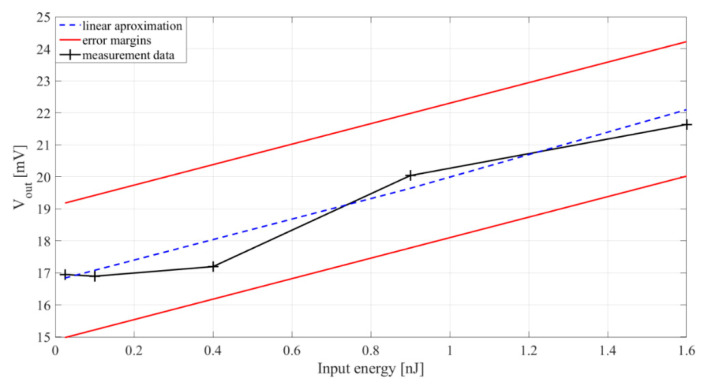
Relation of extractor output voltage (*V_out_*) and input signal energy, *E_in_*. Rectifier capacitances *C_r_*_1−2_ = 33 nF. Inductor *L* = 100 mH. Black pluses—measurement data, blue line—linear interpolation, red lines—error margins.

The experimental characterization of the passive electromechanical feature extractor verifies the simulation results shown in Section 3. Figure 9 shows the transfer characteristic of the PEM FE. The sensitivity of the proposed device is around 2 mV/nJ. As can be seen from the maximal measurement error, the measurement resolution is around ±1 nJ.

**Figure 10:**
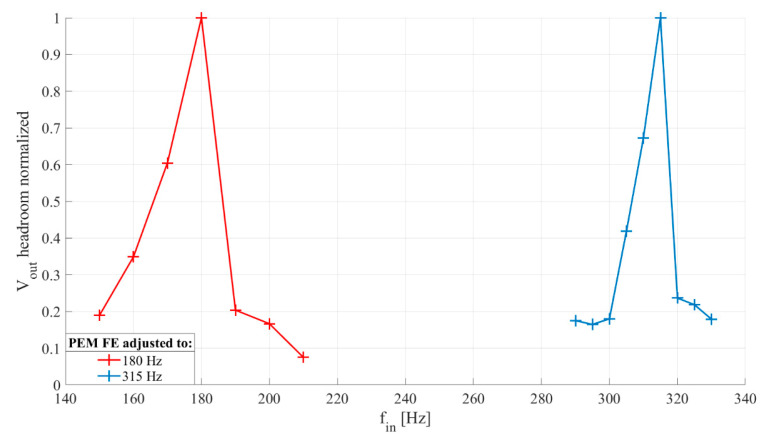
Frequency selectivity of passive electromechanical feature extractor (PEM FE) obtained by change of physical dimensions (beam length (*l*), beam and magnet mass (*m*), magnet position (*δ*)). The output voltage was normalized with regards to maximal value. Input vibrations generate 50 mV peak-to-peak at piezoelectric transducer, input vibration frequency changed by 10 Hz from 150 Hz to 210 Hz and by 5 Hz from 290 Hz to 330 Hz for each developed PEM FE, respectively. Rectifier capacitance *C_r_*_1−2_ = 33 nF, inductor *L* = 100 mH.

Figure 10 shows frequency characteristics of two developed PEM FEs with central frequencies in two different frequency bands of interest for the application in an acoustic detector. This provides verification of the simulation results shown in Figure 5 for three different frequency bands of interest. The number of points in the measured frequency characteristics is lower than in the simulated characteristics for practical reason, and the difference in shapes of the two sets of frequency characteristics is most likely a consequence of the numerical inaccuracies of the simulated model at transitions from the out-of-band to passband frequencies.

**Figure 11:**
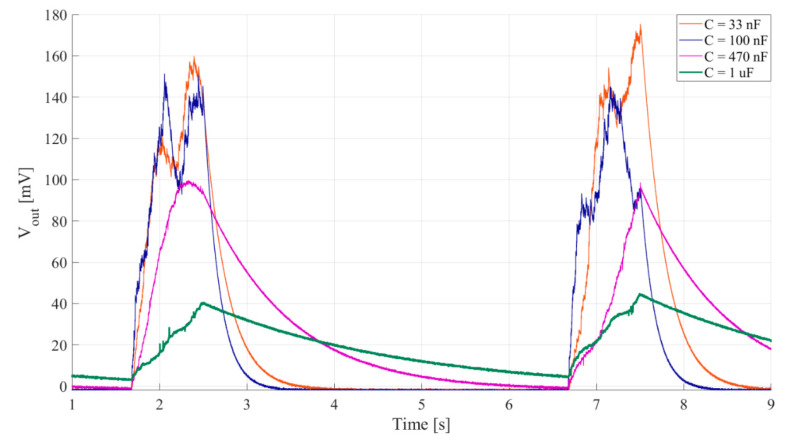
Waveform of the output voltage for different values of rectifier capacitances *C_r_*_1−2_. Voltage generated at the piezoelectric transducer, *V_pzt_*(*t*), is 100 mV peak-to-peak, frequency 315 Hz. Input vibrations are gated, 0.75 s of signal followed by 4 s of pause. Inductor *L* = 100 mH.

It is clear from the waveforms shown in Figure 11 that increasing rectifier capacitances decreases output voltage ripple, but prolongs rise and fall times.

## 5. Comparison of Active Electrical and Passive Electromechanical Feature Extractor

The feature extractors comparison was done using two types of signal: synthetic gated sinusoidal signals with added white noise and a prerecorded speedboat signal.

### 5.1. Measurement Setup

**Figure 12:**
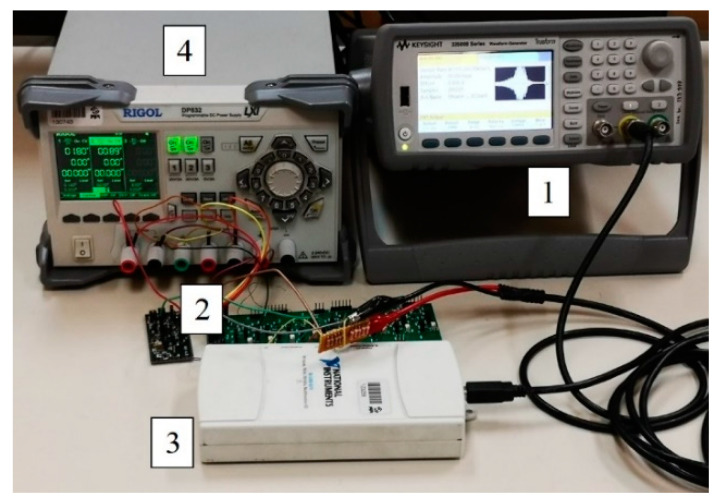
A photograph of the measurement setup for active electrical feature extractor [25,31] measurements. (1) Keysight 33500B waveform generator, (2) active electrical feature extractor, (3) National Instruments (NI) USB-6211 data acquisition card, (4) Rigol DP832 power supply.

The measurement setups for passive electromechanical and active electrical feature extractors are shown in Figure 8a and Figure 12, respectively.

The passive electromechanical feature extractor (PEM FE), shown in Figure 3, was adjusted to generate the highest output voltages at input vibration frequency of 180 Hz. The rectifier capacitors, *C_r_*_1_ and *C_r_*_2_*,* were both set to 1 µF. The inductor, *L*, was set to 100 mH.

The active electrical feature extractor (AE FE) consisted of an active GIC bandpass filter and a passive two-diode voltage doubler, as shown in Figure 1c. The filter central frequency was set to 200 Hz (by *R*_1_, *C_f_*_1_ and *C_f_*_2_), with a pass bandwidth of 200 Hz (set by *R2*, *R*_3_, *C_f_*_1_ and *C_f_*_2_). The rectifier capacitors *C_r_*_1_ and *C_r_*_2_ were both set to 22 nF, to allow the capacitor to fully charge and achieve maximal headroom voltage during each event of interest [25,31].

The measurement setup consisted of the input signal generator (Keysight 33500B), the feature extractor and a data acquisition card (National Instruments NI USB-6211) connected to a PC. The vibrations for the passive electromechanical feature extractor operation were generated by a shaker (Smart Material Energy Harvesting Kit 1.2.) driven by the waveform generator.

### 5.2. Measurement Procedure

The goal of these measurements was to compare the headroom voltages of the active electrical (AE FE) and passive electromechanical feature extractor (PEM FE) in two steps. In the first step, a characterization with synthetic signals of varying amplitudes and signal to noise ratios (SNRs) was performed, while the second step provided verification using a prerecorded speedboat signal.

The vibrations driving the passive electromechanical feature extractor were set to generate peak-to-peak voltage at the piezoelectric transducer, *V_pzt_*, equal to the peak-to-peak voltage at the output of the filter, *V_filt_*, of the active electrical feature extractor, for the same input signal waveform. The voltage amplitudes ranged from 10 mV to 70 mV peak-to-peak, in steps of 10 mV.

#### 5.2.1. Synthetic Signal

**Figure 13:**
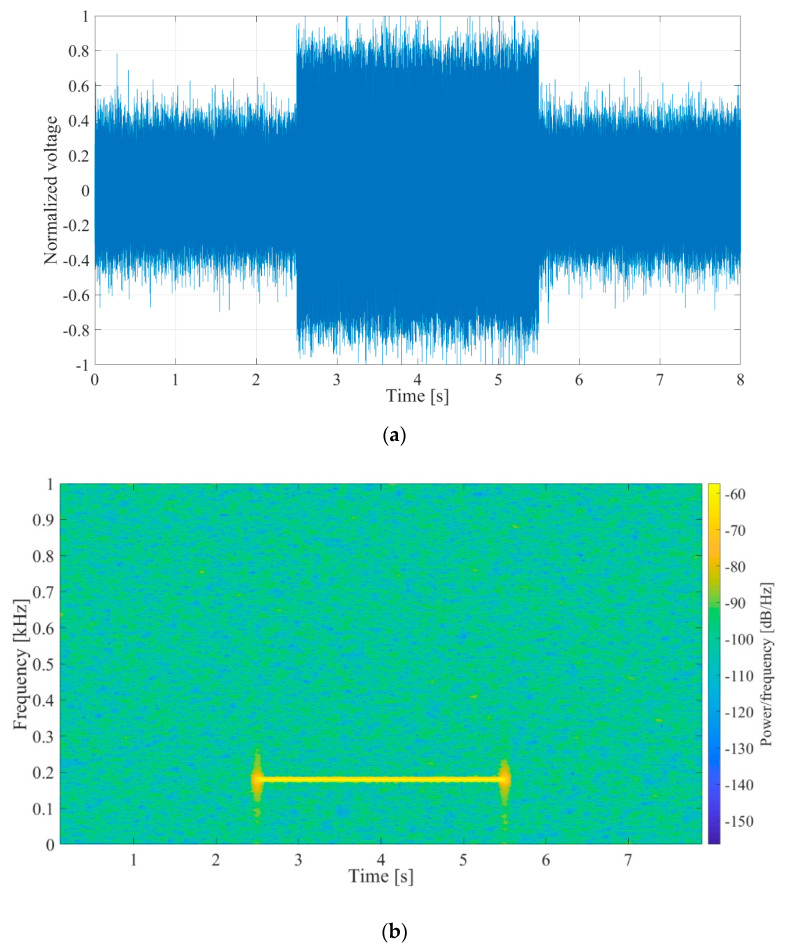
(**a**) Waveform of the synthetic input signal, *V_in_*, 3 s of 180 Hz sinus, followed by 5 s pause, 0 dB signal to noise ratio (SNR). The voltage shown was normalized with regards to maximal value. (**b**) Spectrogram of the synthetic input signal.

The synthetic signal was a gated 180 Hz sinusoidal signal of varying amplitude lasting for 3 s, followed by 5 s of pause. White noise was added to the signal to achieve SNRs of −10 dB, 0 dB and 10 dB. The 0 dB SNR signal waveform with normalized amplitudes and its spectrogram are shown in Figure 13a,b, respectively.

#### 5.2.2. Prerecorded Signal

**Figure 14:**
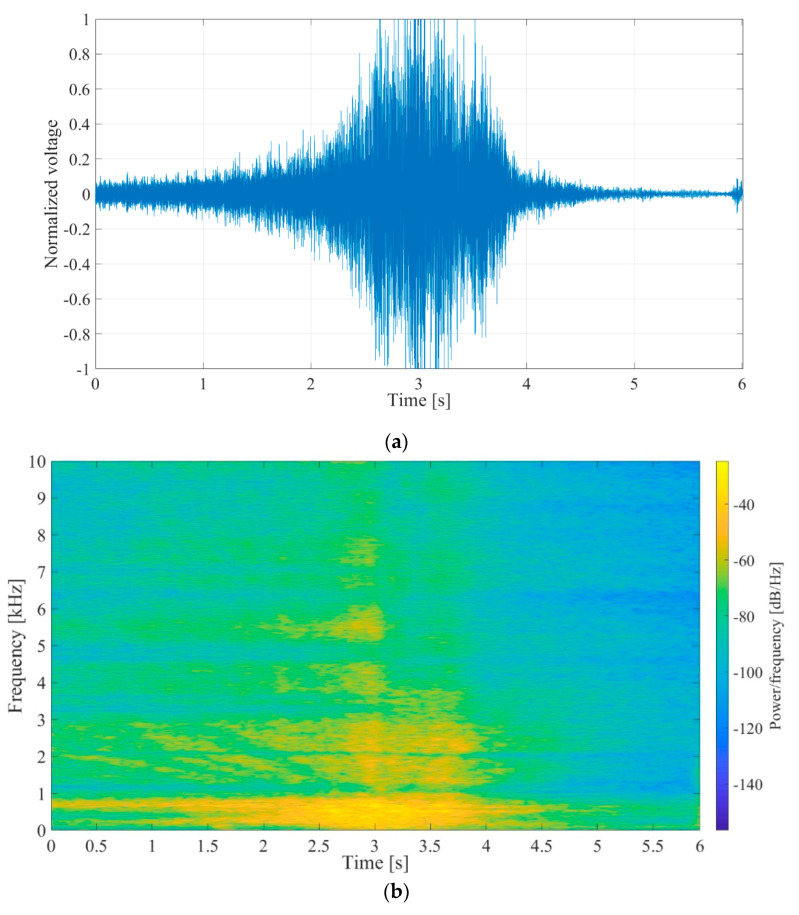
(**a**) Waveform of the prerecorded input signal, *V_in_*, with a duration of approximately 3 s, followed by around 3 s of pause. The voltage shown was normalized with regards to maximal value. (**b**) Spectrogram of the prerecorded input signal.

The prerecorded signal was a signal of a twin-engine speedboat passing over a hydrophone submerged approximately 1 m under the surface in shallow water [36]. The signal waveform with normalized amplitudes and its spectrogram are shown in Figure 14a,b, respectively. Signal duration is approximately 3 s, followed by around 3 s of pause.

### 5.3. Results

**Figure 15:**
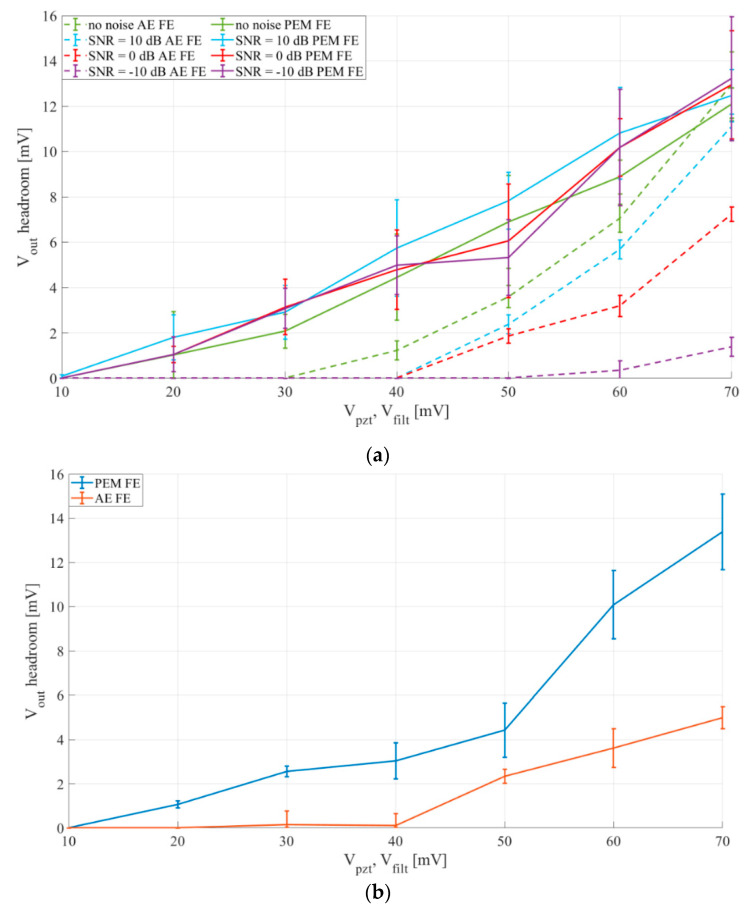
Comparison of outputs of a passive electromechanical feature extractor (PEM FE) and an active electrical feature extractor (AE FE). Rectifier capacitances for AE FE *C_r_*_1−2_ = 22 nF, for PEM FE *C_r_*_1−2_ = 1 µF. PEM FE inductor *L* = 100 mH. (**a**) Synthetic input signals, 3 s of sinus, 180 Hz, 5 s of pause, filter and piezoelectric transducer output, *V_filt_*, *V_pzt_*—10–70 mV peak-to-peak, (**b**) Prerecorded speedboat signal, 3 s of signal, 3 s of pause, filter and piezoelectric transducer output, *V_filt_*, *V_pzt_*—10–70 mV peak-to-peak.

The obtained comparison for synthetic signals and prerecorded speedboat signal are shown in Figure 15a,b, respectively.

The results show that the passive electromechanical feature extractor (PEM FE) outperforms the active electrical feature extractor (AE FE), with inputs up to 70 mV, for both synthetic and prerecorded speedboat signal inputs.

The higher headroom voltages of the PEM FE stem from the increase of rectifier efficiency provided by utilization of the mechanically switched inductor. This increased sensitivity allows a detector utilizing the PEM FE to detect events generating signals under 20 mV peak-to-peak, while a detector using the AE FE could only detect events generating signals of more than 40 mV peak-to-peak.

The results also show that the PEM FE is far more resistant to noise than AE FE, as for the input signal SNR ranging from signal with no noise down to an SNR of −10 dB the PEM FE loses less than 1 mV of headroom voltage, while the AE FE loses more than 10 mV of headroom voltage in the same SNR range.

In addition to the mentioned performance improvements, it should also be emphasized that, unlike the previously developed AE FE, the novel feature extractor is fully passive. The previously developed AE FE consists of an adjustable bandpass filter and an envelope tracker, and consumes 8.25 µW of the total 11.52 µW consumed by each channel [25], meaning that replacing it with a fully passive feature extractor, as proposed in this work, would reduce each channel’s power consumption by around 72%, proving that utilizing this method would either greatly extend the detector’s life-time, or allow us to put in more channels with the same power budget, further increasing the detection accuracy.

## 6. Conclusions

Acoustic infrequent events can be recognized from an ordered sequence of discrete time-frequency states obtained from a low-power multichannel detector comprising filtering, envelope extraction and single bit quantization. The envelope is simple to extract in terms of hardware complexity and power consumption, but the extractor has poor performance in low SNR conditions that are common in undersea environments. The goal of this work was to present and characterize a novel passive electromechanical feature extractor utilizing a mechanically switched inductor, based on RMSHI, as an alternative solution for feature extraction in an acoustic event detector in frequency range from 200 Hz to 1 kHz. Through presented simulation and experimental characterization of the passive extractor we confirmed its applicability for the signals of interest and determined the key design parameters. We demonstrated advantages of the passive electromechanical extractor in comparison to the active electrical version in terms of operation with lower input signal levels (under 20 mV, compared to 40 mV), and higher robustness in low SNR conditions (1 mV output voltage loss compared to 10 mV). In addition to outperforming the signal processing performances, compared to our previous work, utilization of the passive extractor would also decrease the power consumption of a detector’s channel by 72%, enabling life-time extension and/or increased quality of detection with larger number of channels. This work represents the basis for future research towards a fully passive, or self-sustaining infrequent acoustic event detector channel design utilizing an electromechanical device with an emphasized threshold.

## Figures and Tables

**Table 1:** List of used parameters.

Parameter	Unit	Value	Method of Estimation
*m*	kg	0.00082	Model
*d*	kg/s	0.001	Experiment
*δ*	kg m^−2^ s^−2^	300,000	Model
∏	V/m	1.13	Experiment
*τ*	s	162	Literature [27]
